# Oxysterol Signatures Distinguish Age-Related Macular Degeneration from Physiologic Aging

**DOI:** 10.1016/j.ebiom.2018.05.035

**Published:** 2018-06-11

**Authors:** Jonathan B. Lin, Abdoulaye Sene, Andrea Santeford, Hideji Fujiwara, Rohini Sidhu, Marianne M. Ligon, Vikram A. Shankar, Norimitsu Ban, Indira U. Mysorekar, Daniel S. Ory, Rajendra S. Apte

**Affiliations:** a Department of Ophthalmology & Visual Sciences, Washington University School of Medicine, St. Louis, MO, USA; b Neuroscience Graduate Program, Division of Biology and Biomedical Sciences, Washington University School of Medicine, St. Louis, MO, USA; c Diabetic Cardiovascular Disease Center, Washington University School of Medicine, St. Louis, MO, USA; d Department of Medicine, Washington University School of Medicine, St. Louis, MO, USA; e Department of Pathology and Immunology, Washington University School of Medicine, St. Louis, MO, USA; f Center for Reproductive Health Sciences, Department of Obstetrics and Gynecology, Washington University School of Medicine, St. Louis, MO, USA; g Department of Developmental Biology, Washington University School of Medicine, St. Louis, MO, USA

**Keywords:** Age-related macular degeneration, Aging, Lipids, Cholesterol

## Abstract

Macrophage aging is pathogenic in numerous diseases, including age-related macular degeneration (AMD), a leading cause of blindness in older adults. Although prior studies have explored the functional consequences of macrophage aging, less is known about its cellular basis or what defines the transition from physiologic aging to disease. Here, we show that despite their frequent self-renewal, macrophages from old mice exhibited numerous signs of aging, such as impaired oxidative respiration. Transcriptomic profiling of aged murine macrophages revealed dysregulation of diverse cellular pathways, especially in cholesterol homeostasis, that manifested in altered oxysterol signatures. Although the levels of numerous oxysterols in human peripheral blood mononuclear cells and plasma exhibited age-associated changes, plasma 24-hydroxycholesterol levels were specifically associated with AMD. These novel findings demonstrate that oxysterol levels can discriminate disease from physiologic aging. Furthermore, modulation of cholesterol homeostasis may be a novel strategy for treating age-associated diseases in which macrophage aging is pathogenic.

## Highlights

• Age-related macular degeneration (AMD) is a leading cause of blindness in older adults.
• Macrophage aging is pathogenic in numerous diseases of the elderly, including AMD.
• Cholesterol dyshomeostasis is a central feature of aged macrophages.

Although therapies directed against vascular endothelial growth factor (VEGF) have revolutionized the therapeutic landscape for age-related macular degeneration (AMD), these drugs do not work for all patients. In this work, we characterize how innate immune cells known as macrophages change during aging to better understand the mechanisms underlying AMD pathogenesis. Furthermore, we identify the subset of these changes that are a part of healthy aging versus those that contribute to age-associated disease. Our results suggest that plasma levels of 24-hydroxycholesterol may be a candidate biomarker for pathologic macrophage aging.

## 1 Introduction

Age-related macular degeneration (AMD) is a leading cause of blindness in adults over 50 years of age in industrialized nations [1]. Early AMD is characterized by the presence of lipoproteinaceous deposits or drusen under the retinal pigment epithelium (RPE) and/or thickening of Bruch's membrane. Although early AMD is not always associated with vision loss, it is a major risk factor for progression to one of two forms of advanced AMD: a dry form, characterized by death of RPE cells called geographic atrophy (GA) that eventually leads to death of overlying photoreceptors, or wet (neovascular) form, characterized by abnormal vascular proliferation underneath the retina called choroidal neovascularization (CNV). While both advanced dry and wet AMD can cause vision loss, wet AMD accounts for a significant fraction of the vision loss associated with AMD [2] and can often be acute and catastrophic.

The current mainstay therapies for wet AMD focus on combating abnormal angiogenesis by blocking vascular endothelial growth factor (VEGF) with targeted anti-VEGF agents. These treatments stabilize disease in a majority of patients and significantly improve visual outcomes in 30–40% of wet AMD patients [[3], [4], [5]]. However, these therapies often require frequent visits for repeated intraocular injections, which places a significant burden on patients and caregivers [6]. Moreover, repeated intravitreal injections are also associated with risks of their own, such as infection, scleral thinning, and long-term visual acuity loss due to continued atrophy of the RPE and photoreceptors [3, 7]. Perhaps more importantly, anti-VEGF agents do not address the pathophysiology that causes wet AMD [8]. Therefore, there is need for further research to clarify the molecular and cellular mechanisms involved in the transition from physiologic aging to AMD and to understand the pathogenesis underlying the progression from early to wet AMD, which may lead to novel strategies for targeted intervention.

In the past few decades, we and others have demonstrated that macrophages, key cells of the innate immune system, play important roles in the pathogenesis of wet AMD [[9], [10], [11], [12]]. Moreover, it has become increasingly clear that the ability of macrophages to polarize to different activation states is an important factor affecting whether macrophages promote health or disease [13]. Depending on dynamic tissue signals and the surrounding micro-environment, macrophages can polarize to a classical pro-inflammatory (M1-like) phenotype, an alternative anti-inflammatory (M2-like) phenotype, or some intermediate between these two extremes [14]. To further complicate matters, the identity of the specific activators that cause macrophage polarization may also affect the macrophage phenotype [15].

Previously, we reported that aged macrophages tend to skew to the anti-inflammatory M2-like phenotype and are less able to inhibit abnormal angiogenesis [16]. Furthermore, aged macrophages exhibit both impairments in cholesterol efflux [17] and abnormalities in IL-10 and downstream STAT3 signaling pathways that contribute to this age-associated drift towards M2-like polarization [18]. These age-associated impairments in cholesterol efflux and other lipid-related pathways may have mechanistic consequences in disease pathogenesis [19]. This possibility is supported by the fact that polymorphisms in lipid-related genes, such as hepatic lipase (LIPC), ATP-binding cassette transporter member 1 (ABCA1), and cholesterol ester transfer protein (CETP), are associated with advanced AMD [20]. Moreover, drusen, a clinical feature of early AMD, are lipid-rich, further supporting our hypothesis that dysregulated lipid homeostasis contributes to AMD. Despite these advances, the global programmatic changes that occur during macrophage aging need further elucidation. It is also unclear what subset of these changes are associated with physiologic aging or are pathologic and contribute to age-associated disease.

In this study, we sought to delineate the cellular pathways involved in macrophage aging and identify potential markers that may distinguish age-associated changes that are physiologic versus those that promote age-associated disease. Our results suggest that impaired cholesterol homeostasis in macrophages is a central process perturbed during aging and that these changes lead to alterations in oxysterol signatures that can distinguish AMD from physiologic aging. These findings may allow physicians to monitor progression of disease with quantifiable serum markers and may potentially lead to novel therapeutic strategies not only for AMD but also for other age-associated diseases in which alternatively-activated macrophages are pathogenic.

## 2 Materials and Methods

### 2.1 Animals

All animal experiments were approved by the Institutional Animal Care and Use Committee (IACUC) and performed in accordance with the Washington University School of Medicine Animal Care and Use guidelines. We obtained old (i.e., ~18-month-old), female wild-type C57BL/6J mice from the National Institute on Aging (Bethesda, MD) and compared them to strain-matched young (i.e., ~3-month-old), female wild-type C57BL/6J controls. We harvested peritoneal macrophages five days after elicitation with a 2-ml intraperitoneal injection of 4% thioglycollate broth (Sigma-Aldrich, St. Louis, MO). We harvested splenic macrophages by performing positive magnetic cell separation with the PE selection kit (Stem Cell Technologies) and PE anti-F4/80 monoclonal antibody (clone: BM8; eBioscience, Waltham, MA), following manufacturer's instructions. We cultured peritoneal and splenic macrophages in Gibco™ RPMI 1640 medium (Thermo Fisher Scientific, Waltham, MA) supplemented with 10% fetal bovine serum (FBS; Atlanta Biologicals, Flowery Branch, GA) and 1% penicillin-streptomycin (Thermo Fisher Scientific). When indicated, we treated macrophages with 25 or 50 μg/ml of oxidized LDL (oxLDL; Alfa Aesar, Haverhill, MA) for 24 h prior to further analysis.

### 2.2 OCR Measurements

To perform metabolic characterization, we measured the oxygen consumption rate (OCR) of peritoneal macrophages as a surrogate marker for oxidative respiration with the XF96 Extracellular Flux Analyzer (Seahorse Bioscience, North Billerica, MA). In short, we plated peritoneal macrophages in Seahorse XF96 cell culture microplates (Seahorse Bioscience) at 100,000 cells per well. On the morning of the experiment, we washed the cells and replaced the medium with Seahorse assay medium (Seahorse Bioscience) supplemented with 25 mM glucose (Sigma-Aldrich, St. Louis, MO) and 1 mM sodium pyruvate (Thermo Fisher Scientific) and adjusted the pH to 7.4. After incubation in a non-CO_2_ incubator at 37 °C for 1 h, we measured OCR at baseline and after sequential treatment with the following chemicals from the Mito Stress Test kit (Seahorse Bioscience): 3 μM oligomycin, 5 μM carbonyl cyanide 4-(trifluoromethoxy) phenylhydrazone (FCCP), and 1 μM rotenone/antimycin A (rot/AA). Each cycle consisted of 2 min of mixing and a 1-min pause, followed by a 5-min measurement period; we repeated each cycle 3–4 times. We normalized the background of all measurements by subtracting the average OCR of each sample after treatment with rot/AA.

### 2.3 Gene Expression Analysis

We extracted total RNA from peritoneal macrophages with the RNeasy Mini kit (Qiagen) and prepared cDNA with the High-Capacity Reverse Transcription kit (Applied Biosystems), following manufacturer's instructions. We performed quantitative PCR amplification of cDNA using either the TaqMan® probe-based gene expression assay for *p16*^*INK4a*^ (Mm00494449_m1; Applied Biosystems) or custom TaqMan® Array Plates (Applied Biosystems) for lipid-related genes with the assays indicated in Supplemental Table S1. In all cases, we used the ΔΔCT method, normalizing to *Actb*, *18sRNA*, *Gapdh*, or the geometric mean of a combination of these endogenous controls.

### 2.4 Transcriptomic Profiling

We isolated total RNA from peritoneal macrophages with the mirVana kit (Ambion), performed cDNA amplification with the Ovation® Pico kit (NuGEN, San Carlos, CA), and performed target labeling with the Encore® Biotin kit (NuGEN), according to manufacturer's instructions. We then performed whole transcriptome profiling of young and aged peritoneal macrophages using Mouse Gene (MoGene) 1.0 ST arrays processed with Affymetrix Expression Console (v1.3.1.187) at standard settings (RMA background correction, median polish summarization, and quantile normalization) to generate intensity values. We assigned each probeset of the MoGene 1.0 array a detection call of ‘mean + 2 × SD’ of the negative controls. We performed data quality control to identify potential outliers by principal component analysis (PCA) plot and hierarchical clustering, as well as by quality control (QC) metrics (all probeset RLE means > 0.25) in Expression Console. From this QC, we omitted one sample in the young group. We then filtered data by probeset type (“main” in MoGene 1.0) and by detection call (any probeset without a “detected” call in any of the samples was removed). Any probeset without a gene symbol in the MoGene 1.0 data was also removed. 18,066 MoGene 1.0 probesets (from the total of 35,556) were kept for further analysis. We analyzed the data using the R package “limma” and generated gene lists based on *P*-values and false detection rate (FDR) q-values. We performed gene ontology (GO), pathway map, and interactome analyses with MetaCore™ (Clarivate Analytics, Philadelphia, PA). The microarray data are available at the Gene Expression Omnibus (GEO) at NCBI under accession number GSE111382.

### 2.5 Flow Cytometry

We plated peritoneal macrophages on untreated Petri dishes and allowed them to adhere overnight. The next morning, we lifted cells by incubating for 5 min in ice-cold Dulbecco's phosphate-buffered saline (DPBS; Thermo Fisher Scientific) without calcium or magnesium and scraping gently. We next filtered cells through a 40 μm cell strainer, washed them with DPBS, and resuspended them in DPBS containing 5% FBS, 10 mM HEPES, 1 mM EDTA, and TruStain fcX™ antibody (BioLegend, San Diego, CA). We stained 10^5^ to 10^6^ cells with APC anti-mouse CD36 (clone: HM36; BioLegend), PE/Cy7 anti-mouse CD64 (clone: ×54–5/7.1; BioLegend), and PE anti-mouse F4/80 (clone: BM8; eBioscience) antibodies for 20 min on ice. We then washed and resuspended cells in DPBS and acquired data on a BD X-20 or BD LSR II flow cytometer (BD Biosciences, San Jose, CA). We analyzed and visualized data with FlowJo v10.

### 2.6 Human Subjects

This study was approved by the Human Research Protection Office of Washington University School of Medicine and adhered to the Declaration of Helsinki. We obtained informed consent from all subjects prior to blood collection. To purify peripheral blood mononuclear cells (PBMCs) and plasma, we performed density gradient centrifugation with BD Vacutainer CPT™ Cell Preparation Tubes (Franklin Lakes, NJ), following manufacturer's instructions. We classified patients as no AMD, early AMD, or wet AMD based on established clinical criteria [21]. Early AMD patients had either moderate drusen (>63 μm) or pigment changes in at least one eye but no CNV or GA in either eye at the time of sample collection. Wet AMD patients had CNV in at least one eye at the time of sample collection. We stored all samples at −80 °C until further analysis. To determine the appropriate sample size, we performed an a priori power analysis with G*Power 3.1 [22]. Estimating an effect size d of 0.6 based on pilot experiments, we calculated that we needed 94 subjects with an allocation ratio of 1.5:1 to detect a significant difference between the groups at the two-sided α = 0.05 level with 80% power. For the subanalysis, we performed chart review to identify the closest total plasma cholesterol measurement obtained within 3.5 years of the date of sample collection.

### 2.7 Oxysterol Profiling by LC–MS/MS

We extracted and quantified oxysterol levels from murine peritoneal macrophage pellets, the supernatant of murine peritoneal macrophages, human PBMC pellets, and human plasma samples as previously described [23]. Briefly, we added deuterated oxysterols to the samples as internal standards and then extracted oxysterols with methanol. We derivatized the extracted oxysterols and their internal standards with *N*,*N*-dimethylglycinate (DMG) to increase MS sensitivity. We performed oxysterol analysis with a Shimadzu 20 CE HPLC system (Kyoto, Japan) and a LeapPAL autosampler coupled to a tandem mass spectrometer (API 4000; Applied Biosystems) operated in MRM mode. We used positive ion ESI mode for detection of the derivatized oxysterols, injecting study samples in duplicate for data averaging. We conducted data processing with Analyst 1.5.1 (Applied Biosystems) and determined relative levels of each oxysterol by comparing its measurement with that of its corresponding deuterated internal standard. We normalized the murine macrophage oxysterol levels to cell number, murine supernatant levels to volume, human PBMC oxysterol levels to protein content, and human plasma oxysterol levels to volume. To normalize human samples processed on different days, we used one of the patient samples as an internal control.

### 2.8 Statistics

We performed statistical analysis as indicated in the Figure legends with Prism 5 (GraphPad Software) or SPSS Statistics Version 23 (IBM, Armonk, NY). We assessed the normality of our data graphically and with the Kolmogorov-Smirnov test and used appropriate non-parametric alternatives when necessary. We considered *P* < .05 to be statistically significant. To model the relationship between PBMC and plasma oxysterol signatures and AMD, we generated binary logistic regression models. Our model included the following predictor variables: age at the time of sample collection, gender, and PBMC/plasma oxysterol levels. For the subanalysis, we generated an additional binary logistic regression model, which included the following predictor variables: age at the time of sample collection, gender, plasma 24-HC levels, and total plasma cholesterol. For all regression models, we assessed fit with the Hosmer-Lemeshow lack-of-fit test and performed diagnostics by examining Cook's distances, leverages, and residual deviances. The final reported models had seven omitted cases based on sensitivity analysis. We checked for problems with collinearity by examining variance inflation factors (VIF). We used an unadjusted alpha of 0.05 for the binary logistic regression.

## 3 Results

### 3.1 Peritoneal Macrophages from Old Mice Exhibit Features of Aging

Under steady-state conditions, tissue-resident macrophages are maintained through constant replacement or self-renewal. In many tissues, including the peritoneal cavity, there is extensive replacement of macrophages as quickly as every 3 weeks [24]. Therefore, we sought to determine whether macrophages isolated from old (i.e., 18-month-old) wild-type mice exhibited features of cellular aging when compared to macrophages isolated from young (i.e., 3-month-old) wild-type mice despite this short replenishment cycle.

**Fig. 1:**
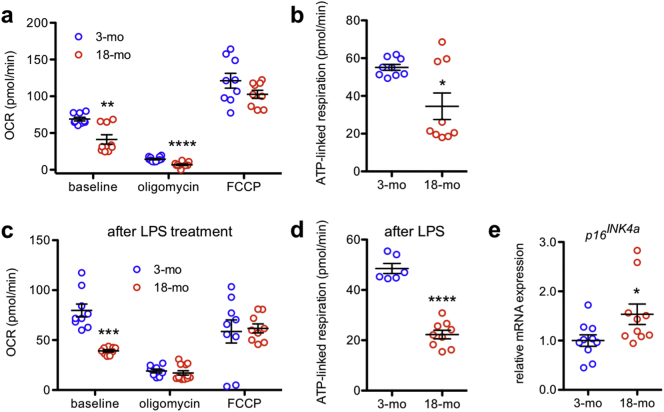
Peritoneal macrophages from old mice exhibit quantifiable signs of aging. (a) Aged peritoneal macrophages had reduced oxygen consumption rate (OCR) both at baseline (*N* = 9/group; 2-tailed, unpaired Welch's t-test) and in response to oligomycin (*N* = 11–12/group; 2-tailed, unpaired t-test) and significantly reduced ATP-linked respiration (b; *N* = 9/group; 2-tailed, unpaired Welch's t-test). (c) Lipopolysaccharide (LPS)-treated aged peritoneal macrophages also had impaired mitochondrial bioenergetics at baseline (*N* = 8–12/group; 2-tailed, unpaired Welch's t-test) and significantly reduced ATP-linked respiration (d; *N* = 6–9/group; 2-tailed, unpaired t-test) compared to LPS-treated young peritoneal macrophages. (e) Aged peritoneal macrophages had increased mRNA expression of the senescence marker *p16*^*INK4a*^ (*N* = 10/group; 2-tailed, unpaired t-test). Open circles depict individual data points; horizontal lines depict mean ± SEM (a-e) (* *P* < .05; ** *P* < .01; *** *P* < .001; **** *P* < .0001).

Mitochondrial dysfunction is a well-established hallmark of aging, and in many tissues, there is an age-dependent decrease in mitochondrial oxygen consumption [25]. To determine whether peritoneal macrophages from old mice exhibit mitochondrial dysfunction, we measured the oxygen consumption rate (OCR) of peritoneal macrophages isolated from 3-month-old and 18-month-old mice. The baseline OCR of peritoneal macrophages from old mice was significantly lower than that of peritoneal macrophages from young mice (Fig. 1a), indicating reduced basal oxidative respiration. Although both young and aged peritoneal macrophages appropriately exhibited a reduction in OCR after treatment with the ATP synthase inhibitor oligomycin, young peritoneal macrophages maintained a somewhat higher residual OCR (Fig. 1a). In contrast, both young and aged peritoneal macrophages demonstrated similar maximal oxidative respiration in response to the uncoupling agent FCCP (Fig. 1a). The difference between the baseline OCR and the OCR after oligomycin treatment represents ATP-linked respiration. Aged peritoneal macrophages exhibited significantly reduced ATP-linked respiration (Fig. 1b), indicating mitochondrial dysfunction.

Moreover, previous studies report that exposing macrophages to lipopolysaccharide (LPS) considerably alters their bioenergetic profile [26]. Furthermore, such a metabolic switch is required for macrophage activation and affects subsequent inflammatory status [[26], [27], [28]]. We observed distinct mitochondrial respiratory profiles when comparing young and aged, LPS-treated peritoneal macrophages: aged peritoneal macrophages exhibited a reduced OCR at baseline, despite maintaining similar responses to oligomycin and FCCP (Fig. 1c). Again, aged peritoneal macrophages exhibited significantly reduced ATP-linked respiration (Fig. 1d). Cumulatively, these data demonstrate that mitochondrial oxidative metabolism is considerably impaired in peritoneal macrophages isolated from old mice both at baseline and in response to LPS.

Furthermore, we evaluated the expression of the aging marker *p16*^*INK4a*^, which is a known senescence marker in bone marrow-derived macrophages [29] and has been shown to accumulate in rodent and human tissues during aging [[30], [31], [32]]. We found that *p16*^*INK4a*^ mRNA expression was significantly elevated in aged peritoneal macrophages compared to young peritoneal macrophages (Fig. 1e). Collectively, these results confirm that despite their constant renewal, peritoneal macrophages from old mice exhibit multiple quantifiable signs of aging, including mitochondrial dysfunction and increased expression of the senescence marker, *p16*^*INK4a*^.

### 3.2 Aged Macrophages Exhibit Impaired Cholesterol Homeostasis

**Fig. 2:**
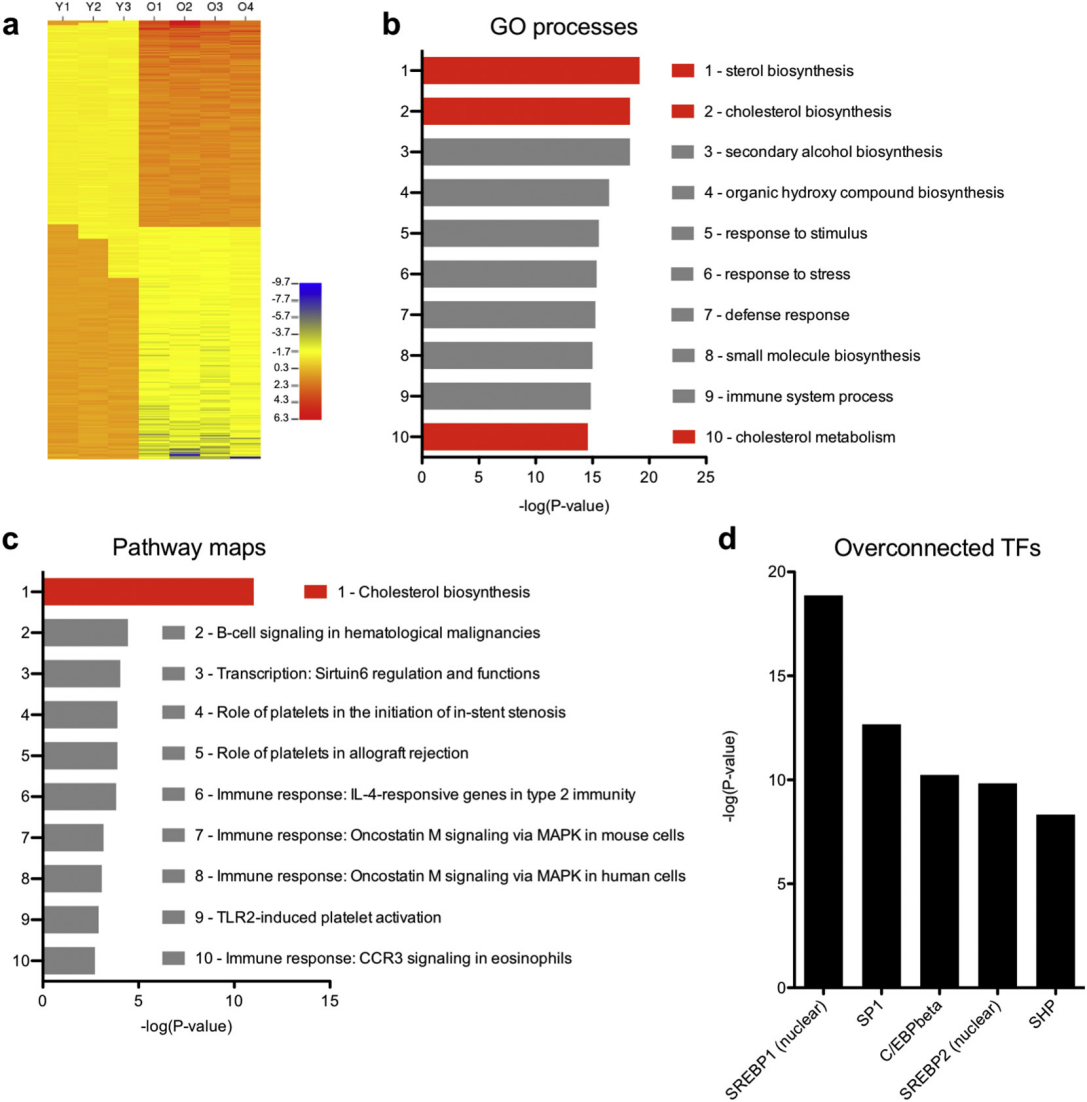
Transcriptomic profiling of aged peritoneal macrophages. (a) Aged peritoneal macrophages display numerous transcriptomic changes, which suggest perturbations in various gene ontology (GO) processes (b) and pathway maps (c). (d) Interactome analysis revealed numerous overconnected transcription factors (TFs) whose known gene targets were overrepresented in the genes we identified as dysregulated in aged versus young macrophages.

To determine the specific cellular processes that are perturbed in aged macrophages, we profiled the transcriptomes of young and aged macrophages with the GeneChip Mouse Gene 1.0 ST Array (Affymetrix, Santa Clara, CA). We found that 1080 probe sets were significantly differentially expressed in aged versus young macrophages (Fig. 2a; *P* < .05, FDR < 0.20). The twenty protein-coding genes that were up- or downregulated with the highest fold change in aged versus young macrophages are presented in Table 1. To determine whether the identities of the dysregulated genes were associated with defects in particular pathways or cellular processes, we filtered for genes with a 1.50-fold-change cutoff (22.1%, *N* = 239) and then performed gene ontology (GO) and pathway map analysis with MetaCore™ (Clarivate Analytics, Philadelphia, PA). Of interest, the first, second, and tenth most significant GO processes implicated were sterol biosynthesis, cholesterol biosynthesis, and cholesterol metabolism, respectively (Fig. 2b). Consistently, cholesterol biosynthesis was the most significant pathway map implicated by the altered transcriptomic profile of aged macrophages (Fig. 2c). As expected, the transcriptomic profile of aged macrophages also suggested disruptions in numerous other immune response pathways (Fig. 2c). Overall, these results clearly indicate that cholesterol homeostasis is significantly perturbed during macrophage aging.

**Table 1:** Top ten up- and down-regulated genes in aged versus young peritoneal macrophages.

Probeset ID	Gene symbol	Fold change^a^	P-value	FDR
10463355	*Scd2*	−3.645	0.0007	0.0667
10506571	*Dhcr24*	−2.522	0.0001	0.0361
10347748	*Acsl3*	−2.406	0.0003	0.0497
10560702	*Ceacam19*	−2.266	<0.0001	0.0307
10403413	*Idi1*	−2.070	0.0018	0.0952
10544273	*Clec5a*	−1.996	0.0006	0.0634
10482762	*Idi1*	−1.977	0.0021	0.1006
10420668	*Mir15a*	−1.969	0.0016	0.0924
10424349	*Sqle*	−1.969	<0.0001	0.0115
10527920	*Cyp51*	−1.967	0.0012	0.0851
10582879	*Csprs*	3.771	<0.0001	0.0258
10538126	*Gimap4*	4.368	0.0052	0.1382
10551025	*Cd79a*	6.435	0.0018	0.0957
10429520	*Ly6d*	6.483	0.0028	0.1104
10466172	*Ms4a1*	7.480	0.0025	0.1050
10392142	*Cd79b*	7.764	0.0004	0.0552
10458278	*Mzb1*	9.573	0.0002	0.0469
10523359	*Cxcl13*	11.166	0.0015	0.0918
10531724	*Plac8*	11.549	0.0006	0.0634
10429564	*Ly6a*	12.571	<0.0001	0.0306

a Aged versus young peritoneal macrophages; negative fold-change reflects decreased expression in aged macrophages relative to young macrophages.

Given that numerous genes were dysregulated in aged peritoneal macrophages, we sought to identify candidate transcription factors (TFs) that may regulate the aging process. Therefore, we performed interactome analysis to identify over-connected TFs, which may regulate the observed transcriptomic changes in aged macrophages. Of interest, two of the top five most significantly overconnected TFs were sterol regulatory element-binding protein 1 and 2 (SREBP1/SREBP2; Fig. 2d), which are known master regulators of cholesterol and lipid homeostasis. These two TFs had connectivity ratios of 14.73 and 15.15, respectively, indicating ~15-fold overrepresentation of their known targets. These findings support our assertion that global lipid homeostatic mechanisms are impaired in aged macrophages.

To determine the specific aspects of cholesterol homeostasis that are impaired in aged macrophages, we analyzed the expression profile of 113 lipid-related genes in young and aged macrophages with a custom quantitative PCR array (Applied Biosystems). We were able to detect expression of 86.7% (*N* = 98) of the genes we tested and found that 30 of these 98 cholesterol-related genes (30.6%) had significantly different expression in aged versus young macrophages (Table 2). When we subdivided these lipid-related genes broadly by their cellular function, we observed that they encompassed diverse biological processes, including cholesterol/lipid biosynthesis, elimination, transport, and uptake, among other processes. These findings once again confirmed that aged peritoneal macrophages exhibit global impairments in their ability to maintain cholesterol and lipid homeostasis.

**Table 2:** Expression profiling of lipid-related genes in aged and young macrophages.

Cellular function	Gene symbol	Fold change^a^	P-value^b^
Biosynthesis	*Fads2*	−1.545	0.050
	*Fads3*	1.299	0.050
	*Fdft1*	−3.831	0.050
	*Fdps*	−1.885	0.050
	*Hmgcs2*	3.122	0.050
	*Prkaa2*	2.773	0.046
	*Scd1*	−1.895	0.050
Elimination	*Cyp11a1*	−9.342	0.050
	*Cyp7b1*	2.515	0.046
	*Cyp27a1*	−1.473	0.050
	*Hadhb*	−1.984	0.050
	*Lpl*	1.496	0.050
	*Tbxas1*	1.249	0.050
Leukotriene signaling	*Lta4h*	−1.314	0.050
	*Alox5ap*	−1.919	0.050
Transport	*Apoa1*	−6.985	0.046
	*Apof*	−6.089	0.050
	*Slc16a6*	−1.459	0.050
	*Slc27a1*	−1.659	0.050
	*Slc27a3*	−4.402	0.050
	*Stard4*	−1.306	0.050
Uptake	*Cxcl16*	−1.927	0.050
	*Olr1*	−2.340	0.050
	*Pcsk9*	−1.903	0.050
	*Stab2*	−3.008	0.046
Other or multiple functions	*Adfp*	2.102	0.050
	*Alox15*	5.797	0.050
	*Fabp4*	1.338	0.050
	*Nr0b2*	−3.911	0.037
	*Nr1h3*	−1.561	0.050

a Aged versus young peritoneal macrophages; negative fold-change reflects decreased expression in aged macrophages relative to young macrophages.

b Significant by 2-tailed Mann-Whitney U test.

### 3.3 Aged Macrophages have Altered Intracellular Oxysterol Content

We previously reported that aged macrophages have higher levels of intracellular cholesterol related to impaired efflux [17], likely related to the global changes in cholesterol homeostatic mechanisms described above. The oxidation of cholesterol to generate oxidized derivatives of cholesterol or oxysterols serves a crucial purpose to facilitate elimination of excess cholesterol. However, oxysterols themselves also play important signaling roles in regulating cholesterol homeostasis [33] and inflammation [34] and may therefore promote disease. In addition, aberrant oxysterol production can be a sign of increased oxidative stress, which is known to be pathogenic in AMD [35]. Since our transcriptomic profiling demonstrated that impaired cholesterol homeostasis is a hallmark feature of aged macrophages, we sought to explore whether these changes were associated with altered oxysterol signatures, which would not only provide mechanistic insight into why there is impaired cholesterol homeostasis but also identify a potential approach for detecting defective cholesterol homeostasis.

**Fig. 3:**
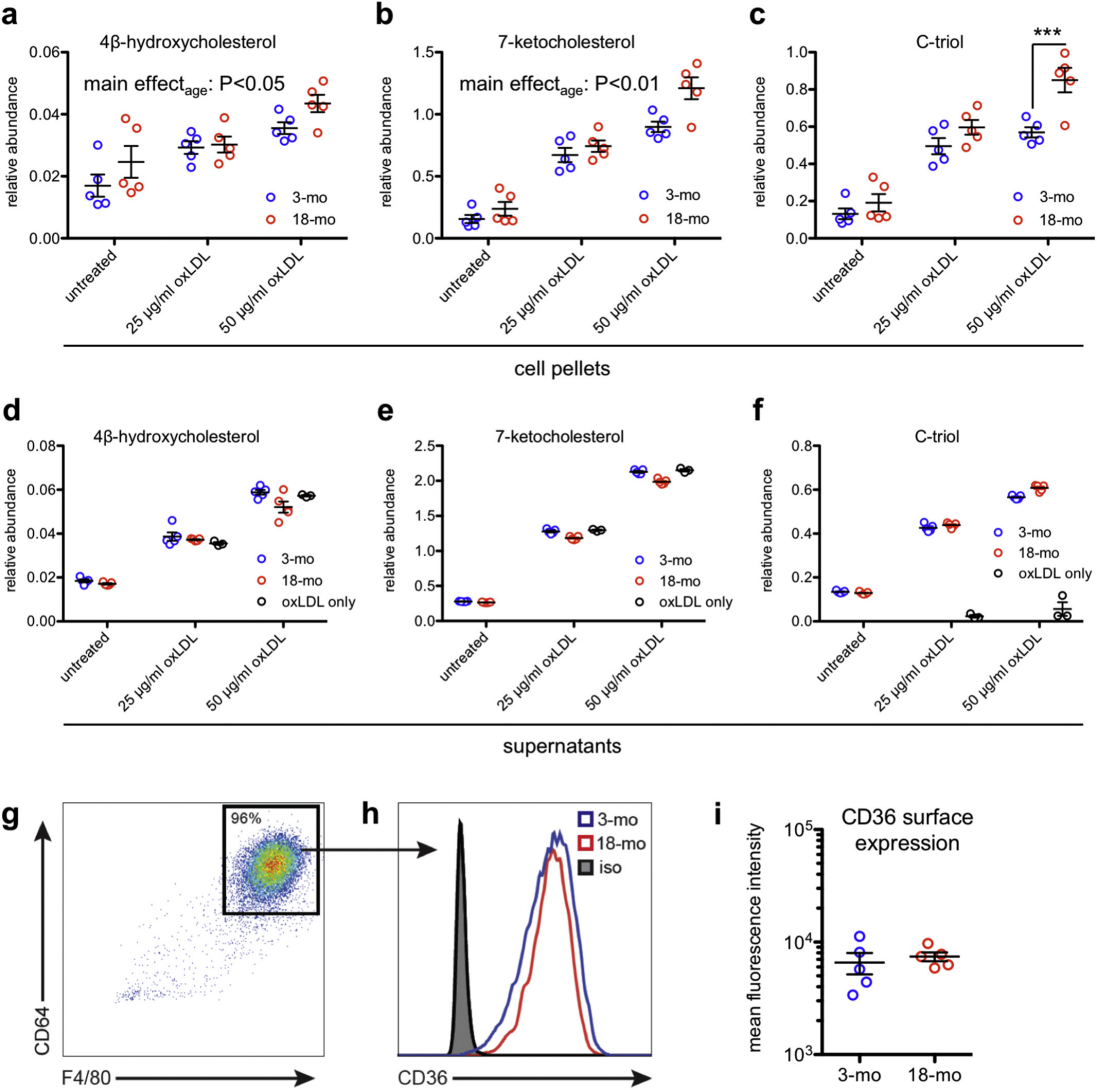
Aged peritoneal macrophages have abnormal oxysterol content. (a-c) Aged peritoneal macrophages contained significantly more intracellular 4β-hydroxycholesterol (4β-HC) and 7-ketocholesterol (7-KC) than their young counterparts at baseline and after treatment with 25 or 50 μg/ml oxidized LDL (oxLDL) (*N* = 5/group; 2-way ANOVA) and significantly more intracellular cholestane-3β,5α,6β-triol (C-triol) after treatment with 50 μg/ml oxLDL (*N* = 5/group; 2-way ANOVA with Bonferroni post-hoc test). (d-f) Although some comparisons were statistically significant due to low within-group variance, the supernatants of young and aged peritoneal macrophages contained qualitatively similar levels of 4β-HC, 7-KC, and C-triol both at baseline and after treatment with oxLDL (*N* = 5/group; 2-way ANOVA with Bonferroni post-hoc test). (g) Representative flow cytometry plot from young and aged peritoneal macrophages showing gating on macrophage markers CD64 and F4/80. (h-i) Young and aged peritoneal macrophages exhibited similar CD36 surface expression. Isotype staining (iso) was identical between groups (*N* = 5/group; 2-tailed, unpaired t-test). Open circles depict individual data points; horizontal lines depict mean ± SEM (a-f, i) (*** *P <* .001).

Using liquid chromatography tandem mass spectrometry (LC–MS/MS), we measured the most abundant oxysterols, including 4β-hydroxycholesterol (4β-HC), 7-ketocholesterol (7-KC), and cholestane-3β,5α,6β-triol (C-triol) in young and aged peritoneal macrophages. We found that aged peritoneal macrophages contained more 4β-HC and 7-KC compared to young peritoneal macrophages, both at baseline and after treatment with 25 or 50 μg/ml oxidized LDL (oxLDL) (Fig. 3a-b). Aged peritoneal macrophages also had increased C-triol content at baseline and after treatment with oxLDL, but the difference was statistically significant only after treatment with 50 μg/ml oxLDL (Fig. 3c). To account for the possibility that these increases in intracellular oxysterols may have been influenced by environmental factors, we also tested a separate cohort of young and aged mice that were housed at the same animal facility. Consistent with our original findings, these aged peritoneal macrophages also had increased levels of 4β-HC, 7-KC, and C-triol after treatment with 50 μg/ml oxLDL (Fig. S1a–c). These findings demonstrate that aged peritoneal macrophages have increased intracellular oxysterols upon challenge with oxLDL and that this difference is likely an effect of age rather than environmental factors.

We also measured the oxysterol content in the supernatant to determine whether increased intracellular oxysterol content was associated with increased oxysterol secretion. Although some comparisons were statistically significant due to low within-group variance, the levels of 4β-HC, 7-KC, and C-triol were qualitatively similar in the supernatant of both young and aged peritoneal macrophages both at baseline and after treatment with oxLDL (Fig. 3d–f; Fig. S1d–f). Of note, the levels of 4β-HC and 7-KC detected in equivalent dilutions of oxLDL were similar to those detected in the supernatants of both young and aged peritoneal macrophages (Fig. 3d–e; Fig. S1d–e), suggesting that any differences in the secretion of these two oxysterols were likely masked by the oxysterols present in the oxLDL itself.

Our finding of increased intracellular oxysterols in the absence of changes in extracellular levels suggested that there is increased oxysterol synthesis associated with aging rather than changes in uptake. In support of this hypothesis, we previously reported that young and aged macrophages have similar capacity to influx Dil-labeled oxLDL [17]. Furthermore, using flow cytometry, we found that surface expression of CD36, a receptor for oxLDL uptake, was similar in young and aged peritoneal macrophages (Fig. 3g–i), supporting the conclusion that these differences in intracellular oxysterols were not likely due to differences in uptake.

To determine whether these aging-associated changes in oxysterol signatures were specific to elicited peritoneal macrophages, we also measured intracellular oxysterol content and secretion in young and aged splenic macrophages. We found that aged splenic macrophages had similar patterns of increased intracellular oxysterols (Fig. S2a–c) with minimal changes in extracellular oxysterols (Fig. S2d–f). These findings indicate that impaired cholesterol homeostasis is associated with detectable alterations in oxysterol signatures in aged macrophages of multiple origins.

### 3.4 PBMC and Plasma Oxysterol Signatures are Altered with Age in Humans

We have previously demonstrated that aged murine macrophages exhibit functional shifts that are associated with their tendency to promote AMD [9, 17, 18]. However, it remains unclear which subset of the age-associated changes described above promote disease rather than being a part of physiologic aging. To discriminate between these physiologic versus pathologic changes, we assessed whether PBMCs and plasma samples from healthy human subjects also exhibited changes in oxysterol signatures with age and whether these changes were different in AMD patients. We chose to process the human samples minimally without cell sorting or cultures to explore how these oxysterol signatures could be used clinically. Demographic information of the human subjects is shown in Table 3.

**Table 3:** Demographic and clinical characteristics of human subjects.

Demographic parameter	Control	AMD	P-value
Age, median (range)	64.09 (21.59–87.07)	74.11 (47.87–100.50)	<.0001^a^
Sex, N			
Male	37	23	.3781^b^
Female	25	22	
AMD status, N			
No AMD	62	0	N/A
Early AMD	0	21	
Advanced neovascular AMD	0	24	

a Significant by 2-tailed Mann-Whitney U test.

b Non-significant by χ^2^ test.

**Fig. 4:**
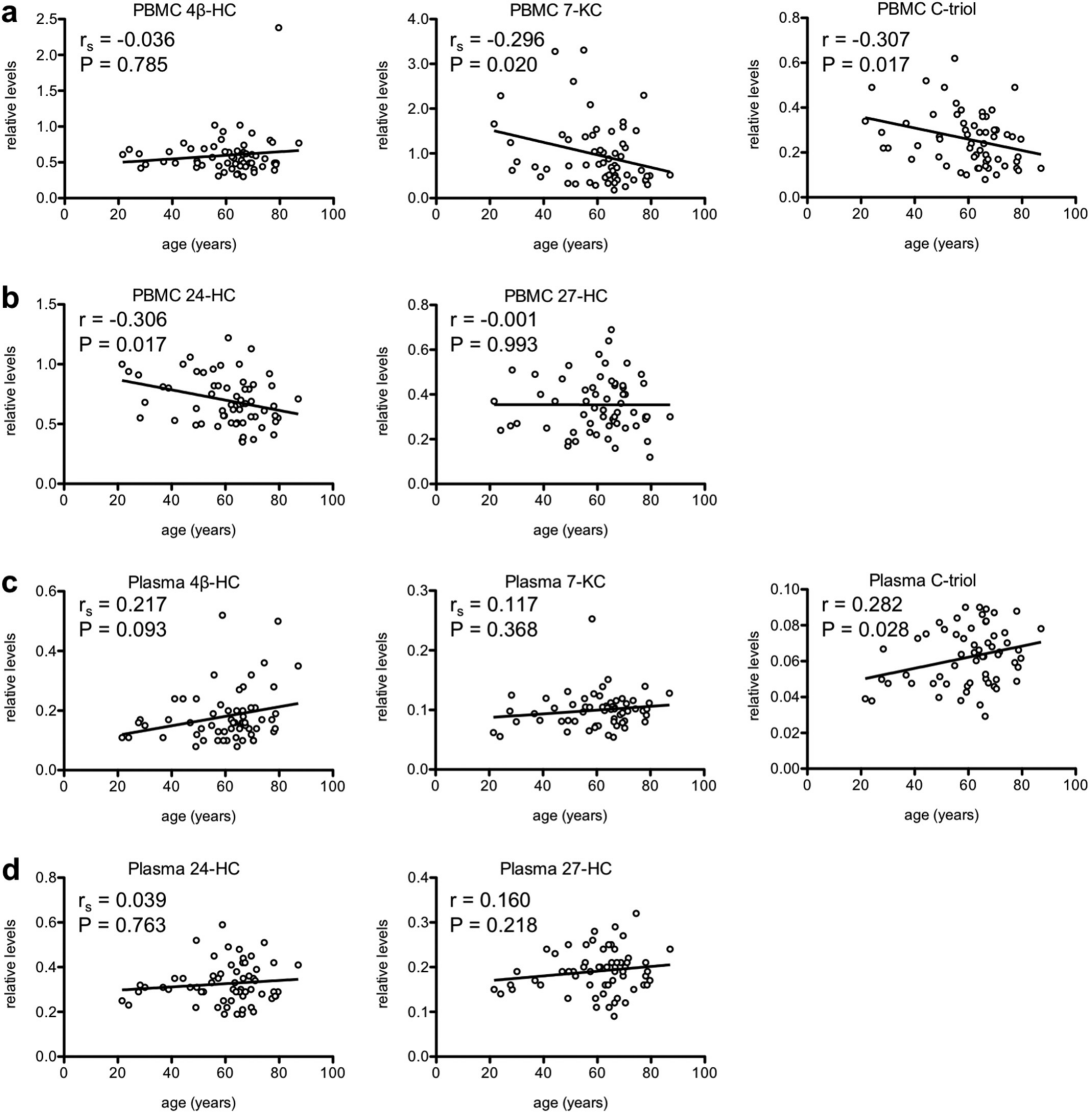
Age affects human peripheral blood mononuclear cell (PBMC) and plasma oxysterol signatures. (a-b) There was a significant negative correlation between age and PBMC 7-KC levels, PBMC C-triol levels, and PBMC 24-HC levels in healthy human subjects. (c-d) There was a significant positive correlation between age and plasma C-triol levels and a trend towards a positive correlation between age and plasma 4β-HC levels. Open circles depict individual data points; lines depict the best-fitting linear regression line (a-d; r = Pearson product-moment correlation coefficient; r_s_ = Spearman rank-order correlation coefficient).

In addition to measuring the same oxysterols we measured in murine peritoneal macrophages (i.e., 4β-HC, 7-KC, and C-triol), we were also able to quantify levels of two additional enzymatically generated oxysterols, 24-hydroxycholesterol (24-HC) and 27-hydroxycholesterol (27-HC), which were below the limit of detection in the murine samples. Of interest, we observed a statistically significant negative correlation between age and PBMC 7-KC levels (Fig. 4a; Spearman *R* = −0.2964, *P* = .0204), PBMC C-triol levels (Fig. 4a; Pearson *R* = −0.3068, *P* = .0171), and PBMC 24-HC levels (Fig. 4b; Pearson *R* = −0.3058, *P* = .0165). There was no association between age and PBMC 4β-HC levels (Fig. 4a; Spearman *R* = −0.0356, *P* = .7853) or PBMC 27-HC levels (Fig. 4b; Pearson *R* = −0.0012, *P* = .9930). Additionally, we observed a significant positive correlation between age and plasma C-triol levels (Fig. 4c; Pearson *R* = 0.2818, *P* = .0278). Although not statistically significant, there was a trend (0.05 < *P* < .10) towards a positive correlation between age and plasma 4β-HC levels (Fig. 4c; Spearman *R* = 0.2170, *P* = .0930). There was no statistically significant correlation between age and plasma 7-KC levels (Fig. 4c; Spearman *R* = 0.1172, *P* = .3683), plasma 24-HC levels (Fig. 4d; Spearman *R* = 0.0394, *P* = .7631), or plasma 27-HC levels (Fig. 4d; Pearson *R* = 0.1601, *P* = .2177). Overall, these findings suggest that healthy humans also exhibit age-associated alterations in oxysterol signatures, likely reflecting changes in cholesterol homeostasis.

### 3.5 Plasma 24-hydroxycholesterol Distinguishes AMD from Physiologic Aging

**Fig. 5:**
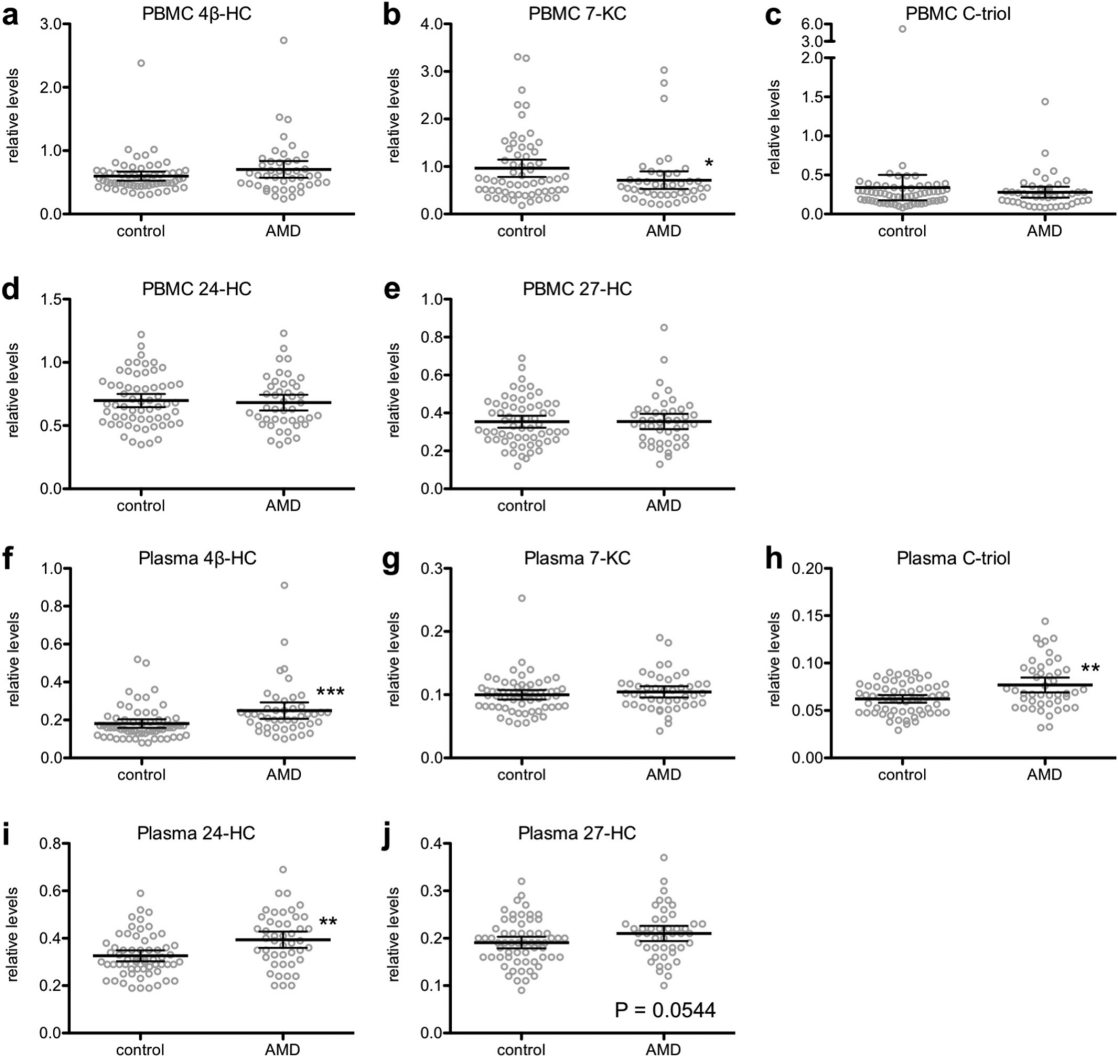
Age-related macular degeneration (AMD) patients have altered peripheral blood mononuclear cell (PBMC) and plasma oxysterol signatures. (a-j) We measured PBMC and plasma levels of 4β-HC, 7-KC, C-triol, 24-HC, and 27-HC. AMD patients (*N* = 44–45) had decreased PBMC 7-KC levels (b; 2-tailed Mann-Whitney U test), elevated plasma 4β-HC levels (f; 2-tailed Mann-Whitney U test), elevated plasma C-triol levels (h; 2-tailed Mann-Whitney U test), elevated plasma 24-HC levels (i; 2-tailed Mann-Whitney U test), and a trend towards elevated plasma 27-HC levels (j; 2-tailed, unpaired t-test) compared to non-AMD controls (*N* = 61). Open circles depict individual data points; horizontal lines depict mean ± 95% confidence intervals (a-j) (* *P <* .05; ** *P <* .01; *** *P <* .001).

To further characterize the relationship between age-associated oxysterol signatures and age-related disease, we recruited human patients with early or advanced neovascular (wet) AMD and measured their PBMC and plasma oxysterol levels (Fig. 5a–j). While AMD patients were significantly older than non-AMD control subjects, there was no difference between the groups on the basis of gender (Table 3). On average, AMD patients had decreased PBMC 7-KC levels compared to non-AMD controls (Fig. 5b) but no differences in the other PBMC oxysterol levels (Fig. 5a/c–e). Moreover, AMD patients had elevated plasma 4β-HC levels (Fig. 5f), elevated plasma C-triol levels (Fig. 5h), elevated plasma 24-HC levels (Fig. 5i), and a trend (0.05 < *P* < .10) towards elevated plasma 27-HC levels (Fig. 5j) compared to non-AMD controls. There was no difference in plasma 7-KC levels (Fig. 5g).

Given the known association between AMD and age and the significant age difference between the two groups, we performed binary logistic regression to precisely model the relationship between PBMC or plasma oxysterol levels and AMD after controlling for age and gender. We selected PBMC 7-KC, plasma 4β-HC, plasma C-triol, and plasma 24-HC levels as candidates for further analysis since these were the oxysterols that were significantly different between AMD patients and controls. The binary logistic regression model for a PBMC oxysterol AMD signature included age, gender, and untransformed PBMC 7-KC (Table 4). The overall model was statistically significant (LR Χ^2^ = 34.0, df = 3, *P* < .001) and had good fit (Χ^2^ = 9.6, df = 8, *P* = .294). As expected, age was significantly associated with AMD (*P <* .001) with a beta coefficient of 0.098, indicating that each additional year of age was associated with increased odds of having AMD (aOR = 1.10; 95% CI = 1.05 to 1.16). However, after controlling for age and gender, PBMC 7-KC levels were not associated with AMD (*P* = .140).

**Table 4:** Beta coefficients from PBMC binary logistic regression model.

Predictor variable	aOR (e^β^)	95% CI of e^β^	P-value
Age	1.103	1.049 to 1.161	<0.001
Female gender	0.893	0.335 to 2.381	0.821
PBMC 7-KC levels, 1-unit	0.351	0.087 to 1.412	0.140

The binary logistic regression model for a plasma oxysterol AMD signature included forced entry of age, gender, and any combination of the candidate plasma oxysterol species through automated forward selection (Table 5). The final model was statistically significant (LR Χ^2^ = 44.9, df = 3, *P* < .001), had good fit (Χ^2^ = 14.2, df = 8, *P* = .078), and included age, gender, and plasma 24-HC levels. As expected, age was significantly associated with AMD (*P <* .001) with a beta coefficient of 0.107, indicating that each additional year of age was associated with increased odds of having AMD (aOR = 1.113; 95% CI: 1.06 to 1.17). Of significant interest, even after controlling for age and gender, 24-HC levels were highly associated with AMD (*P <* .001) with a beta coefficient of 11.327, indicating that each additional 0.1-unit increase in relative plasma 24-HC levels was associated with a 3.10-fold increase in odds of having AMD (95% CI: 1.66 to 5.79).

**Table 5:** Beta coefficients from plasma binary logistic regression model.

Predictor variable	aOR (e^β^)	95% CI of e^β^	P-value
Age	1.113	1.055 to 1.174	<0.001
Female gender	0.390	0.119 to 1.282	0.121
Plasma 24-HC levels, 0.1-unit	3.104	1.66 to 5.79	<0.001

**Fig. 6:**
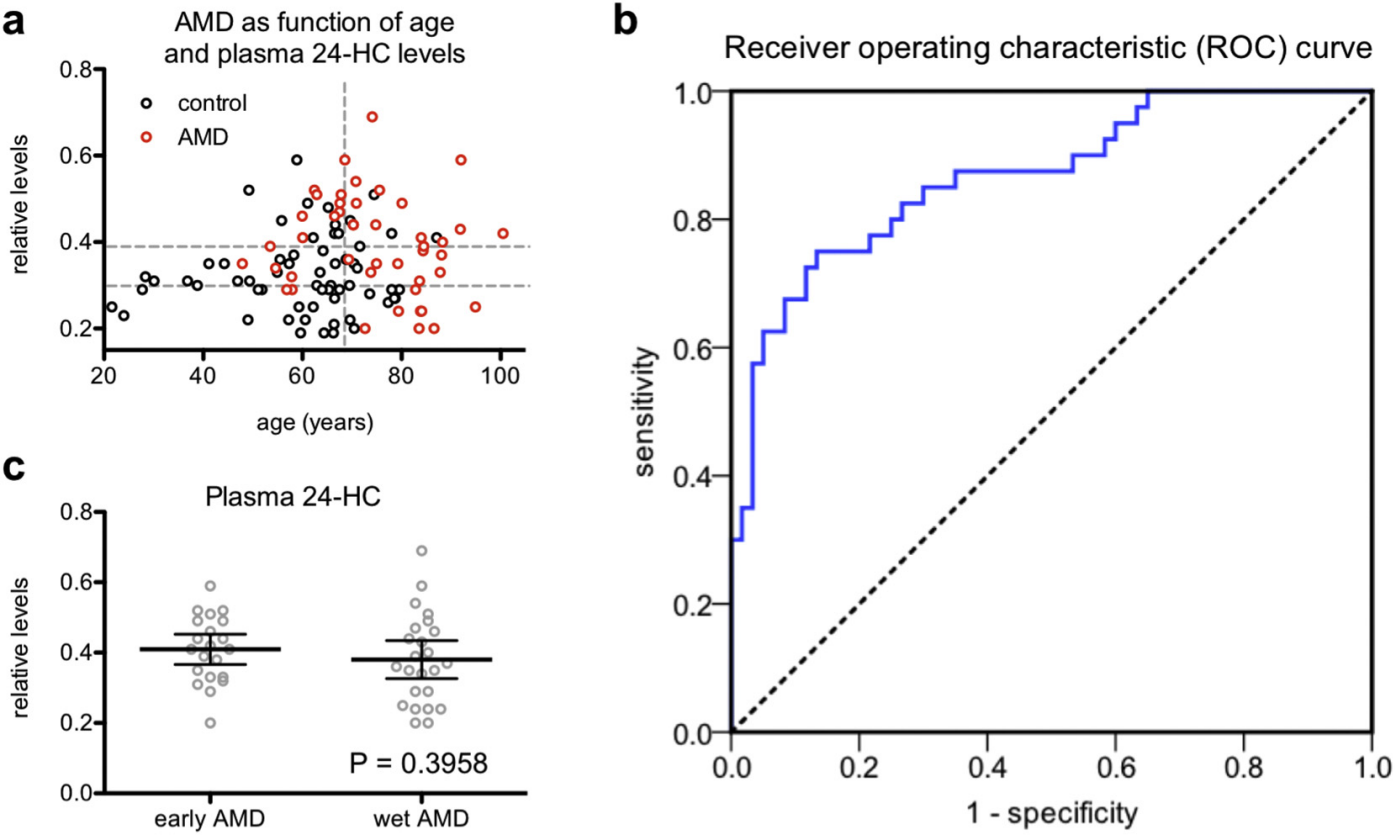
Plasma 24-HC levels discriminate age-related macular degeneration (AMD) from physiologic aging. (a) We divided patients into tertiles by plasma 24-HC and by age (i.e., above versus below median age) and found that individuals in the highest tertile of plasma 24-HC who were also above median age (top right) had the highest AMD prevalence. Horizontal dashed grey lines demarcate plasma 24-HC tertiles; the vertical dashed grey line indicates the median age. (b) Receiver operating characteristic (ROC) curve showing plasma 24-HC discriminates between AMD patients and control subjects. (c) We did not observe a statistically significant difference in plasma 24-HC levels in early AMD patients versus advanced neovascular (wet) AMD patients (*N* = 21 early AMD; 24 wet AMD; 2-tailed, unpaired t-test). Open circles depict individual data points (a, c); horizontal lines depict mean ± 95% confidence intervals (c).

Furthermore, we performed conjunctive analysis to evaluate the clinical utility of using plasma 24-HC to discriminate between AMD patients and non-AMD subjects across varying ages. We divided patients into tertiles by plasma 24-HC levels and by age (i.e., above versus below median), tabulating the AMD prevalence in each conjoined cell (Table 6). This analysis demonstrated a clear stepwise increase in prevalence of AMD in subjects above the median age going from the lowest to the highest tertile of plasma 24-HC (i.e., from 47.1% to 66.7% to 76.2%; Fig. 6a). We observed a similar increase in AMD prevalence in subjects below the median age based on plasma 24-HC tertile (i.e., from 10.5% to 19.0% to 38.5%). These findings support the notion that plasma 24-HC levels can distinguish between the changes in cholesterol homeostasis expected during physiologic aging versus those that suggest risk of AMD. To determine the efficacy of plasma 24-HC levels as a potential marker for AMD, we generated a receiver operating characteristic (ROC) curve and found that the area under the ROC curve (AUC) was 0.866 (95% CI: 0.793 to 0.939), indicating good discrimination (Fig. 6b).

**Table 6:** AMD as a function of age and plasma 24-HC levels.

		Age		
		Below median	Above median	Total
Plasma 24-HC	Lowest tertile	2/19 (10.5%)	8/17 (47.1%)	10/36 (27.8%)
	Middle tertile	4/21 (19.0%)	10/15 (66.7%)	14/36 (38.9%)
	Highest tertile	5/13 (38.5%)	16/21 (76.2%)	21/34 (61.8%)
	Total	11/53 (20.8%)	34/53 (64.2%)	45/106 (42.5%)

To determine the clinical utility of plasma 24-HC levels compared to existing clinical measures of lipid homeostasis, we next analyzed whether plasma 24-HC levels were correlated with total plasma cholesterol levels in the subjects for whom this information was available. In these subjects (*N* = 37), plasma 24-HC was indeed correlated with total plasma cholesterol (*R* = 0.659, *P <* .001). We therefore performed a subanalysis by generating a binary logistic regression for the outcome of AMD with forced entry of plasma 24-HC levels, total plasma cholesterol, age, and gender. This model was statistically significant (LR Χ^2^ = 27.2, df = 4, *P <* .001) and had good fit (Χ^2^ = 2.6, df = 7, *P* = .921). Of interest, total plasma cholesterol levels were not associated with AMD (aOR = 1.014; 95% CI: 0.97 to 1.06; *P* = .547). Remarkably, even after controlling for total plasma cholesterol, age, and gender, plasma 24-HC remained associated with AMD (*P* = .044) with a beta coefficient of 19.308, indicating that each additional 0.1-unit increase in relative plasma 24-HC levels was associated with a 6.90-fold increase in odds of having AMD (95% CI: 1.05 to 45.09). Despite the limited sample size available for this subanalysis, these findings suggest that human plasma 24-HC has strong diagnostic value compared to the existing measure of total plasma cholesterol.

Given the heterogeneity of the clinical progression of AMD and our interest in identifying factors that may predict transition to advanced disease, we also examined whether plasma 24-HC levels differed significantly when subdividing AMD patients into early AMD and advanced neovascular AMD patients. We did not detect a statistically significant difference in plasma 24-HC levels in early AMD versus advanced neovascular AMD patients (Fig. 6c). Therefore, although 24-HC may be associated with AMD, it does not appear to have utility for predicting disease progression based on this cross-sectional analysis.

## 4 Discussion

In this study, we identified that despite their self-renewal, macrophages from old mice exhibit signs of aging, such as defects in mitochondrial oxidative respiration, that are not observed in macrophages from young mice. Consistent with these functional changes, aged macrophages exhibit an altered transcriptomic profile, especially in genes involved in cholesterol homeostasis. These findings build on our previous study reporting that *Abca1* expression is significantly reduced in aged macrophages, leading to impaired cholesterol efflux [17]. We further demonstrate that impairments in cholesterol homeostatic mechanisms are not restricted solely to efflux but globally affect myriad pathways, including biosynthesis, elimination, transport, and regulation. Interestingly, aged macrophages exhibited simultaneous downregulation and upregulation of different genes having similar functions in cholesterol-related pathways (Table 2), indicating broad and complex dysregulation of cholesterol homeostasis.

Our hypothesis that impaired cholesterol metabolism in aged macrophages contributes to AMD pathogenesis is consistent with numerous epidemiological studies that have established that parameters related to lipid status, such as baseline high-density lipoprotein-cholesterol (HDL-C) and total serum cholesterol, affect risk of AMD development and progression [[36], [37], [38]]. Moreover, one past genome-wide association study reported that polymorphisms in lipid-related genes such as LIPC, ABCA1, and CETP are associated with advanced AMD [20]. These findings have led to the hypothesis that statins, cholesterol-lowering drugs used for cardiovascular disease, may affect the development or progression of AMD [39]. The findings of one non-randomized study suggest that high-dose statins may indeed reduce some high-risk features of AMD [40], although large randomized studies are necessary to confirm these findings [41] given that numerous other studies report conflicting results [42]. Cumulatively, these conflicting findings highlight that the relationship between aging, impaired cholesterol homeostasis, and AMD is complex and warrants further investigation.

In this study, we found that in aged murine macrophages, a transcriptomic profile suggesting impaired cholesterol homeostasis was associated with aberrant intracellular oxysterol levels, especially when the macrophages were treated with oxLDL. In many immune cells, oxysterols can directly regulate liver X receptor (LXR) transcriptional activity, which can modulate cellular lipid metabolism and the immune response, especially in inflammation-associated diseases [33]. For example, 27-HC, the most prevalent oxysterol in atherosclerotic lesions, has been shown to promote atherosclerosis by inducing inflammation [43]. Therefore, in addition to being a surrogate marker for impaired cholesterol homeostasis, altered oxysterol production may itself play a pathogenic role in promoting cholesterol dysregulation. Further studies are necessary to uncover the mechanisms underlying altered oxysterol content, which may be related to altered uptake or synthesis. However, our results broadly suggest that altered oxysterols in macrophages are markers of cholesterol dyshomeostasis.

We found that, similar to murine macrophages, human PBMC and plasma samples demonstrated age-dependent and disease-dependent changes in oxysterols. Although the directionality of these changes did not correspond perfectly between mice and humans, our data suggest that broad dysregulation of cholesterol homeostasis in both aging and disease is associated with altered oxysterol signatures. These findings build on recent reports that monocytes isolated from patients with neovascular AMD do indeed exhibit an altered immune-related transcriptomic signature [44] and that these cells, when activated into macrophages, demonstrate proangiogenic characteristics that may contribute to disease pathogenesis [45]. Past studies have also shown that in the outer retina, accumulation of cholesterol oxidation products such as 7-KC can disrupt the immune environment and transform resident macrophages into disease-promoting cells [46]. Specifically, uptake of 7-KC in microglial cells results in decreased production of neurotrophic growth factors and increased expression of angiogenic mediators that promote pathologic CNV [46]. Although there are likely some differences between the mechanisms of altered oxysterols in mice and humans, our work support the broad idea that oxysterols can be used to identify features of cholesterol dyshomeostasis.

How to distinguish whether changes in oxysterol signatures define aging, disease, or both is of great interest, as defects in lipid metabolism are a shared feature of multiple diseases. As an example, certain oxysterols and their metabolites have been shown to be specific biomarkers for Niemann-Pick type C (NPC) disease, an inherited lysosomal storage disease [47, 48]. In this study, we found that after controlling for age and gender, plasma 24-HC was significantly associated with AMD. Despite our modest sample size (*N* = 107), these findings suggest that 24-HC is a strong candidate for an oxysterol that distinguishes AMD from physiologic aging. Although limited by sample size and therefore a relatively wide confidence interval, our subanalysis revealed that plasma 24-HC remained associated with AMD even after controlling for total plasma cholesterol, highlighting its diagnostic value. Larger prospective studies are necessary to validate these findings and identify additional markers of risk.

Cumulatively, our findings highlight that impaired cholesterol homeostasis is a key pathway perturbed in aged macrophages and that oxysterol signatures in patient samples can distinguish AMD from physiologic aging. Ultimately, these findings may not only improve our ability to diagnose disease but also identify targets in cholesterol homeostasis pathways for novel therapeutic approaches.

## Funding Sources

This work was supported by NIH Grants R01 EY019287 (R.S.A.), R01 NS081985 (D.S.O), R01 AG052494 (I.U.M.), P30 EY02687 (Vision Core Grant), and P30 DK020579 (Diabetes Research Center Metabolomics Core); the Starr Foundation (R.S.A.); the Carl Marshall Reeves and Mildred Almen Reeves Foundation (R.S.A.); the Bill and Emily Kuzma Family Gift for retinal research (R.S.A.); a Physician-Scientist Award and a Nelson Trust Award from Research to Prevent Blindness (R.S.A.); the Jeffrey Fort Innovation Fund (R.S.A.); and the Thome Foundation (R.S.A.). Additional funding comes from an unrestricted grant to the Department of Ophthalmology and Visual Sciences of Washington University School of Medicine from Research to Prevent Blindness. J.B.L. was supported by the Washington University in St. Louis Medical Scientist Training Program (NIH Grant T32 GM007200), the Washington University in St. Louis Institute of Clinical and Translational Sciences (NIH Grants UL1 TR002345, TL1 TR002344), and the VitreoRetinal Surgery Foundation. M.M.L. was supported by NIH Grants T32 GM007200 and T32 AI007172. The funders had no involvement in any aspect of this study or manuscript preparation. R.S.A. had full access to all of the data in the study and had final responsibility for the decision to submit for publication.

## Declaration of Interests

The authors declare that no conflict of interest exists.

## Author Contributions

A. Sene performed most of the experiments related to Fig. 1, Fig. 2; J.B.L. performed most of the experiments related to Fig. 3, Fig. 4, Fig. 5, Fig. 6.

Conceptualization: R.S.A., A. Sene, J.B.L.

Investigation: J.B.L., A. Sene, A. Santeford, H.F., R.S., M.M.L., V.A.S., N.B.

Writing – Original Draft: J.B.L., A. Sene

Writing – Review & Editing: J.B.L., A. Sene, R.S.A., D.S.O., N.B., M.M.L., H.F., V.A.S.

Supervision: R.S.A., D.S.O., I.U.M.

Funding Acquisition: R.S.A., D.S.O., I.U.M.

## Appendix A Supplementary data

Supplementary material Image 1
