# Supplementary material for: Oxysterol Signatures Distinguish Age-Related Macular Degeneration from Physiologic Aging
Source: eBioMedicine. 2018 Jun 11;32:9–20. doi: 10.1016/j.ebiom.2018.05.035 (PMC6021272; doi:10.1016/j.ebiom.2018.05.035)
Supplement: Supplementary file 1 — Supplementary material [file mmc1.pdf]

Supplemental Data

Oxysterol signatures distinguish age-related macular degeneration from physiologic aging

Jonathan B. Lin[†], Abdoulaye Sene[†], Andrea Santeford, Hideji Fujiwara, Rohini Sidhu, Marianne M. Ligon, Vikram A. Shankar, Norimitsu Ban, Indira U. Mysorekar, Daniel S. Ory, & Rajendra S. Apte[*]

[†] Co-first authors
[*] Corresponding author

Inventory of Supplemental Information
Supplemental Table S1
Supplemental Figures S1-S2

Supplemental Table S1. Lipid-related gene assays used in custom array plate.

Gene target	TaqMan® assay probe
18sRNA	Hs99999901_s1
Actb	Mm00607939_s1
Abca1	Mm00442646_m1
Abca2	Mm00431553_m1
Abcg1	Mm00437390_m1
Acaa2	Mm00624282_m1
Akr1d1	Mm01165275_m1
Angptl3	Mm00803820_m1
Ankra2	Mm00499234_m1
Apoa1	Mm00437569_m1
Apoa2	Mm00442687_m1
Apoa4	Mm00431814_m1
Apob	Mm01545156_m1
Apoc3	Mm00445670_m1
Apod	Mm00431817_m1
Apoe	Mm00437573_m1
Apof	Mm00506066_g1
Apol8	Mm01195284_m1
Cdh13	Mm00490584_m1
Cel	Mm00486975_m1
Cnbp	Mm00488938_m1
Colec12	Mm01236238_m1
Cxcl16	Mm00469712_m1
Cyb5r3	Mm00504077_m1
Cyp11a1	Mm00490735_m1
Cyp39a1	Mm00517066_m1
Cyp46a1	Mm00487306_m1
Cyp51	Mm00490968_m1
Cyp7a1	Mm00484152_m1
Cyp7b1	Mm00484157_m1
Dhcr24	Mm00519071_m1
Dhcr7	Mm00514571_m1
Ebp	Mm00468191_m1
Ela3/Cela3b	Mm00840378_m1
Fdft1	Mm00815354_s1
Fdps	Mm00836315_g1
Hdlbp	Mm00505507_m1
Hmgcr	Mm01282499_m1
Hmgcs1	Mm01304569_m1
Hmgcs2	Mm00550050_m1
Idi1	Mm01337454_m1

Idi2	Mm00841471_g1
Il4	Mm00445259_m1
Insig1	Mm00463389_m1
Insig2	Mm00460121_m1
Lcat	Mm00500505_m1
Ldlr	Mm00440169_m1
Ldlrap1	Mm00521157_m1
Lep	Mm00434759_m1
Lipe	Mm00495359_m1
Lrp10	Mm00499125_m1
Lrp12	Mm01168065_m1
Lrp1b	Mm00466712_m1
Lrp6	Mm00999795_m1
Lrpap1	Mm00660272_m1
Mbtps1	Mm00490600_m1
Mvd	Mm00507014_m1
Mvk	Mm00445773_m1
Npc1l1	Mm01191972_m1
Nr0b2	Mm00442278_m1
Nr1h4	Mm01240553_m1
Nsdhl	Mm00477897_m1
Olr1	Mm00454586_m1
Osbpl1a	Mm00498552_m1
Osbpl5	Mm00600357_m1
Pcsk9	Mm01263610_m1
Pmvk	Mm01212763_m1
Ppard	Mm00803184_m1
Prkaa1	Mm01296700_m1
Prkaa2	Mm01264789_m1
Prkag2	Mm00513977_m1
Scap	Mm01250183_g1
Scarf1	Mm01240399_m1
Snx17	Mm01220541_g1
Soat1	Mm00486279_m1
Soat2	Mm00448823_m1
Sorl1	Mm01169562_m1
Srebf1	Mm00550338_m1
Srebf2	Mm01306292_m1
Stab1	Mm00460390_m1
Stab2	Mm00454684_m1
Stard3	Mm00445520_m1
Tm7sf2	Mm01233546_g1
Trerf1	Mm01278040_m1

Vldlr	Mm00443281_m1
Zmynd15	Mm02600120_g1
Acat1	Mm00507463_m1
Acadvl	Mm00444296_m1
Adfp/Plin2	Mm00475794_m1
Alox12	Mm00545833_m1
Alox15	Mm01250458_m1
Alox5	Mm01182743_m1
Alox5ap	Mm00802100_m1
Cd36	Mm00432403_m1
Gapdh	Mm99999915_g1
Cyp27a1	Mm00470430_m1
Fabp4	Mm00445878_m1
Fabp5	Mm00783731_s1
Fads1	Mm00507605_m1
Fads2	Mm00517221_m1
Fads3	Mm00517643_m1
Gyk	Mm00433896_m1
Hadhb	Mm00523880_g1
Lta4h	Mm01246216_m1
Ltc4s	Mm00521864_m1
Lpl	Mm00434770_m1
Nr1h3	Mm00443451_m1
Pla2g4a	Mm00447040_m1
Pparg	Mm01184322_m1
Slc16a6	Mm00506192_m1
Slc27a1	Mm00449511_m1
Slc27a3	Mm01220009_g1
Stard4	Mm00505395_m1
Scd1	Mm00772290_m1
Tbxas1	Mm00495553_m1
Ucp2	Mm00627599_m1

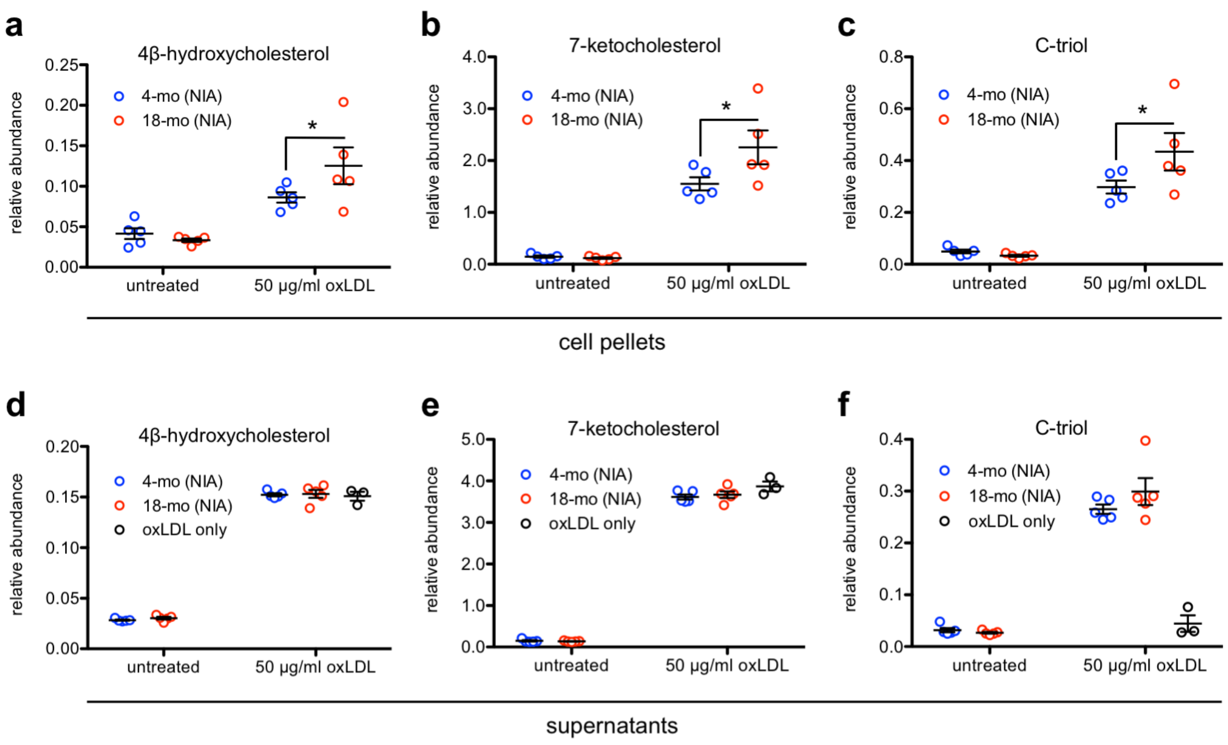

Supplemental Figure S1. Aged peritoneal macrophages have abnormal oxysterol content compared to young peritoneal macrophages isolated from mice housed at the same facility. (a-c) Aged peritoneal macrophages contained significantly more intracellular 4β-hydroxycholesterol (4β-HC), 7-ketocholesterol (7-KC), and cholestane-3β,5α,6β-triol (C-triol) than their young counterparts after treatment with 50 µg/ml oxidized LDL (oxLDL) (N = 5/group; 1-tailed Mann-Whitney U test). (d-f) The supernatant of young and aged peritoneal macrophages contained similar levels of 4β-HC, 7-KC, and C-triol both at baseline and after treatment with oxLDL (N = 5/group; 2-tailed Mann-Whitney U test). Open circles depict individual data points; horizontal lines depict mean ± S.E.M. (a-f) (* P < .05).

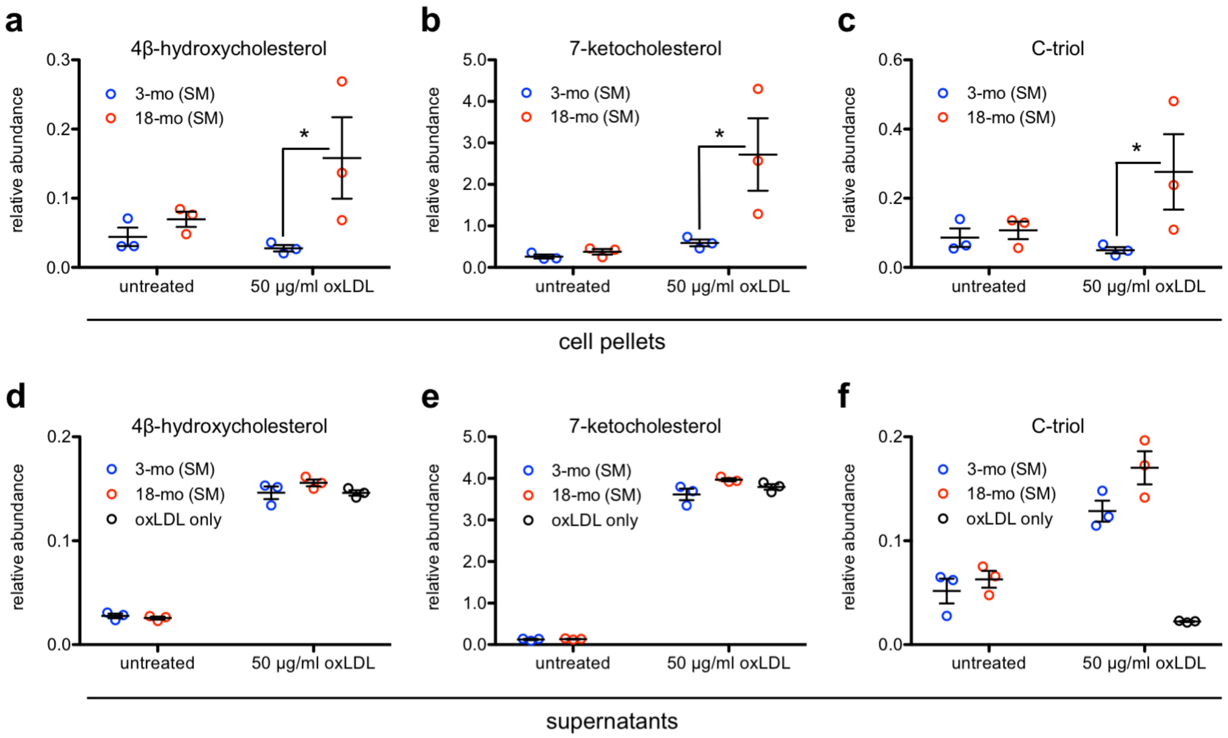

Supplemental Figure S2. Aged splenic macrophages (SM) have abnormal oxysterol content compared to young SM. (a-c) Aged SM contained significantly more intracellular 4β-hydroxycholesterol (4β-HC), 7-ketocholesterol (7-KC), and cholestane-3β,5α,6β-triol (C-triol) compared to their young counterparts after treatment with 50 µg/ml oxidized LDL (oxLDL) (N = 3/group; 1-tailed Mann-Whitney U test). (d-f) The supernatant of young and aged SM contained similar levels of 4β-HC, 7-KC, and C-triol both at baseline and after treatment with oxLDL (N = 3/group; 2-tailed Mann-Whitney U test). Open circles depict individual data points; horizontal lines depict mean ± S.E.M. (a-f) (* P < .05).
